# Early Six-Minute Walk Test May Predict Midterm Outcomes Following Coronary Artery Bypass Grafting

**DOI:** 10.21470/1678-9741-2022-0459

**Published:** 2023-06-14

**Authors:** Hayanne O. Pauletti, Walter José Gomes, Isadora S. Rocco, Marcela Viceconte, Bruna Caroline Matos Garcia, Natasha O. Marcondi, Caroline B. Bublitz, Ariele dos Santos Costa, Thâmara Pequeno de Paiva, Giovanna Domingues Spina, Isis Begot, Célia Camelo Silva, Rita Simone L. Moreira, João Nelson Rodrigues Branco, Guilherme Flora Vargas, Nelson A. Hossne Jr., Ross Arena, Solange Guizilini

**Affiliations:** 1 Programa de Pós-Graduação em Cardiologia, Universidade Federal de São Paulo, São Paulo, São Paulo, Brazil; 2 Disciplina de Cirurgia Cardiovascular, Hospital São Paulo, Escola Paulista de Medicina, Universidade Federal de São Paulo, São Paulo, São Paulo, Brazil; 3 Departamento de Ciências do Movimento Humano, Faculdade de Fisioterapia, Universidade Federal de São Paulo, Santos, São Paulo, Brazil; 4 Department of Physical Therapy, College of Applied Health Sciences, University of Illinois at Chicago, Chicago, Illinois, United States of America

**Keywords:** Myocardial Revascularization, Functional Capacity, Postoperative Complications, 6-Minute Walk Test

## Abstract

**Objective:**

This study aims to investigate the ability of the six-minute walk distance (6MWD) as a prognostic marker for midterm clinical outcomes three months after coronary artery bypass grafting (CABG), to identify possible predictors of fall in 6MWD in the early postoperative period, and to establish the percentage fall in early postoperative 6MWD, considering the preoperative baseline as 100%.

**Methods:**

A prospective cohort of patients undergoing elective CABG were included. The percentage fall in 6MWD was assessed by the difference between preoperative and postoperative day (POD) five. Clinical outcomes were evaluated three months after hospital discharge.

**Results:**

There was a significant decrease in 6MWD on POD5 compared with preoperative baseline values (percentage fall of 32.5±16.5%, P<0.0001). Linear regression analysis showed an independent association of the percentage fall of 6MWD with cardiopulmonary bypass (CPB) and preoperative inspiratory muscle strength. Receiver operating characteristic curve analysis revealed that the best cutoff value of percentage fall in 6MWD to predict poorer clinical outcomes at three months was 34.6% (area under the curve = 0.82, sensitivity = 78.95%, specificity = 76.19%, P=0.0001).

**Conclusion:**

This study indicates that a cutoff value of 34.6% in percentage fall of 6MWD on POD5 was able to predict poorer clinical outcomes at three months of follow-up after CABG. Use of CPB and preoperative inspiratory muscle strength were independent predictors of percentage fall of 6MWD in the postoperative period. These findings further support the clinical application of 6MWD and propose an inpatient preventive strategy to guide clinical management over time.

**Table:** 

Abbreviations, Acronyms & Symbols				
6MWD	= Six-minute walk distance		ICU	= Intensive care unit
6MWT	= Six-minute walk test		LITA	= Left internal thoracic artery
ATS	= American Thoracic Society		LVEF	= Left ventricular ejection fraction
AUC	= Area under the curve		MEP	= Maximal expired pressure
BMI	= Body mass index		MIP	= Maximal inspired pressure
CABG	= Coronary artery bypass grafting		MV	= Mechanical ventilation
CI	= Confidence interval		OPCABG	= Off-pump CABG
COPD	= Chronic obstructive pulmonary disease		PEEP	= Positive end-expiratory pressure
CPB	= Cardiopulmonary bypass		POD	= Postoperative day
FiO₂	= Fraction of inspired oxygen		SE	= Standard error

## INTRODUCTION

Coronary artery bypass grafting (CABG) is the standard of care for patients with advanced coronary artery disease, affording clinically significant benefits^[1-3]^. However, several factors following surgery, including general anesthesia, sternotomy, cardiopulmonary bypass (CPB), pleurotomy, and pleural drain insertion, lead to deterioration of pulmonary function and submaximal exercise capacity^[4-6]^. The functional capacity decline early after CABG is unavoidable and inexorable.

Functional capacity, defined as the ability to perform sustained physical activity at submaximal intensity, can be measured by the 6-minute walk test (6MWT)^[7]^. A significant decrease in six-minute walk distance (6MWD) has been reported after CABG compared with preoperative values^[8-11]^. The sequential change in 6MWD (∆6MWD) has been used to evaluate the clinical impact of interventions. Nevertheless, previous research assessing the absolute value of 6MWD did not consider confounding factors, such as weight and step length. Moreover, there is a gap in the literature to investigate whether the relative change in 6MWD would be correlated with worse clinical outcomes after discharge.

Thus, we hypothesize that applying the percentage change distance may be more appropriate to explore the prognostic significance of 6MWD after CABG. Our study aimed: 1) to investigate the ability of the 6MWD as a prognostic marker for midterm clinical outcomes three months after CABG and to identify possible predictors of fall in 6MWD in the postoperative period; and 2) to establish the percentage fall in postoperative 6MWD, considering the preoperative baseline as 100%.

## METHODS

This prospective cohort was evaluated at the Hospital Universitário of the Universidade Federal de São Paulo (São Paulo, Brazil), according to the Strengthening the Reporting of Observational Studies in Epidemiology (or STROBE) guidelines. The Institutional Human Ethics Committee approved the protocol (CAAE: 55700716.7.0000.5505), and written informed consent was obtained from all patients.

### Patients

Patients aged between 35 and 75 years undergoing elective on-pump CABG and off-pump CABG (OPCABG) were recruited for this study. We excluded patients who: 1) were unable to perform any part of the protocol assessment; 2) were diagnosed with chronic or acute pulmonary disease or acute renal failure; 3) presented with neurologic or orthopedic conditions that would impede completion or any part of the protocol assessment; or 4) sustain hemodynamic instability or severe arrhythmias during the protocol assessment. Chronic obstructive pulmonary disease (COPD) patients confirmed by pulmonary function testing according to American Thoracic Society (ATS) standards was also excluded^[12]^.

### Anesthesia and Surgical Procedure

All patients received the same anesthetic technique - induction with midazolam and maintenance with sufentanil and isoflurane (0.5-1%) - and were mechanically ventilated with a tidal volume of 8 ml/kg and a respiratory rate adapted to maintain normocapnia, with a 0.5 fraction of inspired oxygen (FiO2) without positive end-expiratory pressure (PEEP). Administration of intraoperative fluids was dictated by hemodynamic status at the discretion of the anesthetist.

Both CABG techniques (on and off-pump) were performed through a median sternotomy, using the left internal thoracic artery (LITA) complemented with additional saphenous vein grafts. LITA was harvested in a skeletonized fashion. In OPCABG, temporary occlusion of the coronary artery was achieved using a proximal tourniquet of 4-0 polypropylene thread passed through a malleable silicone tube. Subsequently, depending on the graft, side clamping of the ascending aorta was achieved to perform the proximal anastomosis. An Octopus® 3 (Medtronic, Inc®) suction stabilizer was utilized in all cases. In on-pump CABG, CPB was established with cannulation of the ascending aorta and venous drainage through right atrial single cannula, after systemic heparinization with 400 UI/kg, to keep activated clotting time > 480 seconds. Myocardial protection was achieved using intermittent anterograde hypothermic sanguineous cardioplegia, associated with moderate hypothermia (30°C).

A curved soft tubular polyvinyl chloride drain was inserted and exteriorized at the subxiphoid region and positioned in the left costophrenic sinus. In all patients, a straight mediastinal drain was also placed via subxiphoid approach.

### Postoperative Management

All patients were transferred to the intensive care unit (ICU) with orotracheal intubation, ventilated with a FiO₂ to keep arterial oxygen saturation > 90%, tidal volume at 8 mL/kg of predicted body weight, and PEEP of 5 cmH₂O. Patients were extubated according to the ICU protocol. The drains (mediastinal and/or pleural) were routinely removed on postoperative day (POD) 2. All patients underwent the same analgesic protocol administered during the postoperative period (100 mg of tramadol chlorhydrate, four times daily). During all in-hospital PODs, patients were evaluated by the same physiotherapist, and all patients underwent the same cardiac rehabilitation protocol until hospital discharge (*i.e.*, breathing exercises and early ambulation).

### Six-Minute Walk Test

Functional capacity was assessed by the maximum distance achieved during the 6MWT according to the ATS guidelines^[7]^. The predicted distance was calculated based on the prediction equation^[13]^. The test was performed by the same physiotherapist preoperatively (within 48 hours of unit admission) and, necessarily, on POD5. Criteria of interruption according to ATS guidelines were respected to prevent any complications related to the test application.

### Predictors of Percentage Fall in 6MWD

The association between percentage fall in 6MWD was investigated with several variables, including age, body mass index, left ventricular ejection fraction, respiratory muscle strength, operation time, CPB use, number of grafts, mechanical ventilation (MV) time, and ICU length of stay. Respiratory muscle strength was also obtained preoperatively and on POD5 and was quantified by measurement of maximal inspired pressure (MIP) and maximal expired pressure (MEP) using an analog manovacuometer (Comercial Médica®, Brazil). The protocol was performed as described by the ATS^[14]^, and normative reference values were calculated using equations proposed by Neder et al.^[15]^. This measurement was always performed by the same physiotherapist. MV time and the length of ICU and overall postoperative hospital stay were recorded.

### Midterm Clinical Outcomes

Outpatient three-month follow-up was conducted to assess midterm outcomes including angina recurrence, myocardial infarction (considered when there is a change in creatine kinase-myocardial band five times above normal and troponin, and a presence of new “Q” wave with duration ≥ 0.04 seconds), sternum infection (clinically diagnosed by chest tomography), signs of cardiac decompensation (considered when there were hypotension, severe ventricular arrhythmias, variable blocks, or elevation of ST follow-up in multiple leads), and rehospitalization. Therefore, composite midterm outcomes at three-month follow-up were composed by the occurrence of angina recurrence, myocardial infarction, sternum infection, signs of cardiac decompensation, and rehospitalization.

### Statistical Analysis

Categorical data were expressed in absolute (n) and relative (%) terms and continuous variables as mean and standard deviation. Normality distribution of variables was tested by applying the Shapiro-Wilk test. The categorical data were analyzed by the chi-square test.

The 6MWD was obtained, and values were expressed as a percentage of the baseline value, considering the preoperative baseline as 100%. Variables comparing pre- *vs.* postoperative values were evaluated by paired Student’s *t*-tests. A linear multivariable analysis was performed, and the independent predictors for percentage fall in 6MWD in the postoperative were determined by a stepwise method.

Midterm outcomes at three-month follow-up were evaluated as composite outcomes by the occurrence of angina recurrence, myocardial infarction, sternum infection, signs of cardiac decompensation, and rehospitalization.

A receiver-operating characteristic analysis was performed to calculate the area under the curve (AUC) and to indicate prognostic performance of percentage fall in 6MWD with regard to the occurrence of adverse clinical outcomes within the three months tracking period following CABG. The best cutoff value was defined as the highest true positive rate plus true negative rate (sensitivity + specificity) point. A statistical significance level of *P*<0.05 was applied in our study. The IBM Corp. Released 2013, IBM SPSS Statistics for Windows, version 22.0, Armonk, NY: IBM Corp. software was used to perform this analysis.

## RESULTS

**Fig. 1:**
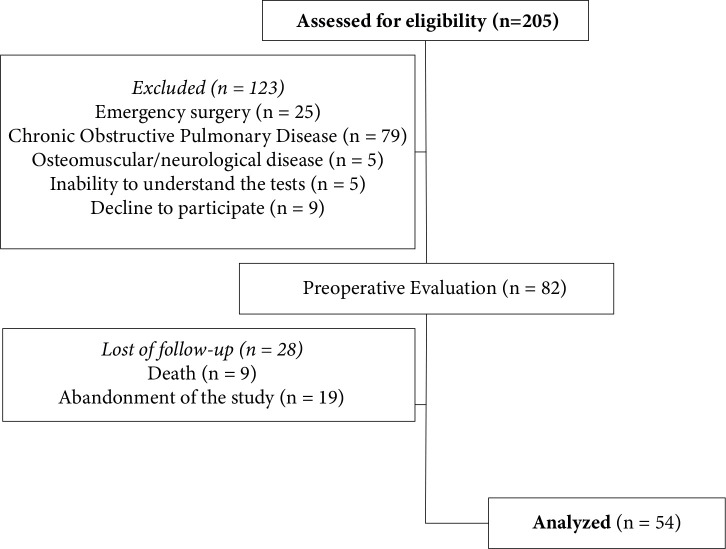
Flowchart of progression of patients through the study period.

Initially, 205 patients were screened for inclusion in this study. Ninety-two patients fulfilled the inclusion criteria, and 54 completed the evaluation. A flow chart indicating the progression of patients throughout the study period is illustrated in Figure 1. Clinical and demographic data are listed in Table 1.

**Table 1:** Characteristics of the study patients.

Variables	N=54
Age (years)	59.9±6.4
Sex (male/female)	50/4
BMI (kg/m^2^)	25.7±2.8
LVEF (%)	
< 45% (n = 26)	36.5±7.4
> 45% (n = 28)	65.0±7.2
6MWD (m)	432.5±86.9
% predicted	76.4±15.1
CABG	
Off-pump (%)	53.8
On-pump (%)	46.2
Pump time (min)	86.3±24.7
Grafts per patient (n)	2.2±0.8
Operation time (min)	263.5±62.6
MV time (hours)	8.8±7.5
Length of hospital stay (days)	
ICU	5.1±1.7
Postoperative period	8.5±3.5
Data are shown as mean ± standard deviation	

6MWD=six-minute walk distance; BMI=body mass index; CABG=coronary artery bypass grafting; ICU=intensive care unit; LVEF=left ventricular ejection fraction; MV=mechanical ventilation

In relation to functional capacity, a significant fall in 6MWD was observed on POD5 considering the preoperative baseline value as 100% (32.5±36.8% of fall, *P*<0.001) (Table 2). All patients also presented with a significant impairment in MIP and MEP on POD5 compared to the preoperative baseline (15.7±7.3% of fall, *P*<0.001) (Table 2).

**Table 2:** Functional evaluation during preoperative period and on POD5.

Variables	Preoperative period	POD5	% Fall
6MWD (m)^a^	432.5±86.9	281.7±139.3^**^	32.5±36.8
Interruption, n (%)^b^	4 (7.4)	16 (29.6)^*^	-
|MIP| (cmH2O)^a^	99.3±26.2	83.8±18.8^*^	15.7±7.3
MEP (cmH2O)^a^	108.5±18.7	90.5±28.7^*^	13.8±6.6

a Paired *t*-test; bMcNemar test to contingency table analysis of paired samples;

* *P*<0.05;

** *P*<0.01 Data are shown as mean ± standard deviation 6MWD=six-minute walk distance; MEP=maximum expired pressure; MIP=maximum inspired pressure; POD5=postoperative day five

Table 3 demonstrates the predictors of percentage fall in 6MWD in the postoperative period. Using linear multiple regression analysis determined by stepwise method, independent predictors of percentage fall in 6MWD on POD5 were CPB use and preoperative inspiratory muscle strength (MIP).

**Table 3:** Determinants of percentage fall in six-minute walk distance in the postoperative period.

	Coefficient	SE	95% CI	*t*-value	*P*-value
Intercept	87.319	14.072	59.011 -115.629	6.205	0.0001
CBP use	19.242	7.013	5.134 - 33.351	2.744	0.009
Preoperative |MIP| (cmH2O)	-0.609	0.131	-0.873 - -0.345	-4.640	0.0001

SE=standard error; Stepwise linear regression model. R^2^=0.40 CI=confidence interval; CPB=cardiopulmonary bypass; MIP=maximal inspired pressure;

Adverse outcomes observed at three months of follow-up were angina recurrence (9.2%), myocardial infarction (1.9%), wound infection (5.5%), signs of heart failure decompensation (5.5%), and rehospitalization (16.7%).

**Fig. 2:**
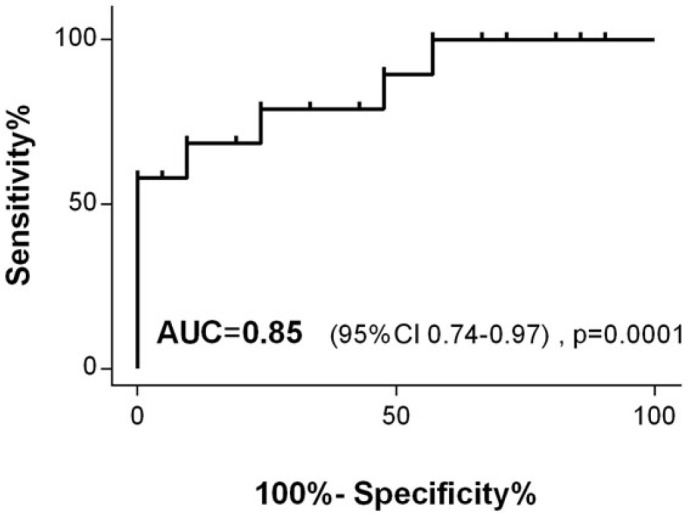
Receiver operating characteristic curve comparing the percentage fall in six-minute walk distance in the postoperative period and adverse clinical outcomes rate in three months (composite outcomes). AUC=area under the curve; CI=confidence interval.

ROC analysis demonstrated that the optimal cutoff value of percentage fall in 6MWD to predict events within three months of follow-up was 34.6% (AUC = 0.85, sensitivity = 78.95%, specificity = 76.19%, *P*=0.0001) (Figure 2) with a positive predictive value of 75% and a negative predictive value of 80%.

## DISCUSSION

The primary finding of the current study was the establishment of a cutoff value where patients who exhibit > 34.6% fall in 6MWD presented with poorer outcomes within three months following CABG. This finding suggests a potential application of an early 6MWT as a tool to predict midterm outcomes after CABG and possibly guide preventive therapeutic management.

Exercise capacity and tolerance are essential in providing clinical and prognostic insight in patients with cardiovascular disease^[16]^. Functional capacity assessed by the 6MWT has extensive acceptance due to convenience, low cost, and presumed ease of completion. The 6MWT can be performed by a broad spectrum of patients, including those who are severely limited, such as cardiac patients after recent major surgery^[17]^.

The functional capacity decline after CABG is unavoidable and inexorable. The dependence of distance walked from several demographic, anthropometric, clinical, and physiological factors underlines the need for expressing the results of 6MWT both as an absolute value in meters and as a percent-predicted value, according to an established reference equation^[14,18]^. However, when evaluating patients undergoing a surgical procedure, the result expressed as a percentage of an individual’s baseline value can be more appropriate. Our study evaluated the percentage fall in 6MWD during postoperative period, using the preoperative assessed value as 100%.

We found an average fall of 32.5% in 6MWD on POD5 compared to the baseline preoperative value. Similarly, some studies performed the 6MWT during the preoperative and early postoperative periods. However, the assessments were performed on different PODs, leading to different results. Cordeiro et al.^[19]^ (2016) observed a drop in 6MWD of 16% performing the test at approximately POD8. Hirschhorn et al.^[9]^ (2012) found a decline in 6MWD of 12.3% performing the test at approximately POD7, and Guizilini et al.^[10]^ (2014) observed a fall in 6MWD of 22% performing the test on POD6. In this current study, the 6MWT was applied necessarily on POD5, and a higher decline value in the percentage fall in 6MWD, observed in our results, may be due to the earlier application (POD5) of the test following surgery compared to the abovementioned studies. Considering the early postoperative period in conjunction with the current common practice of early hospital discharge^[20,21]^, we performed the evaluation on POD5 to standardize a reference period to verify the impact of percentage fall in 6MWD on outcomes within three months of CABG. Also, POD5 was chosen based on previous cutoff value of the prolonged length of ICU stay, in order to early detect severe functional capacity impairment and enable the perioperative heart team to reconsider rehabilitation strategies still during hospitalization^[22]^.

Many factors can contribute to this impairment in functional capacity during the postoperative period, including prolonged bed rest, pain, and respiratory limitations after sternotomy. Previous studies have shown impairment of pulmonary function (^i.e.^, decreased lung volumes and capacities in the postoperative period due to general anesthesia, CPB use^[17]^, diaphragmatic dysfunction, and use of LITA with pleurotomy and pleural drainage^[10,16,23]^). The pulmonary dysfunction associated with chest pain and the leg incision for vein harvesting may have influenced the lower performance in the 6MWT in the early postoperative period.

In this current study, linear regression analysis demonstrated that CPB use and preoperative MIP were the determinants of percentage fall in 6MWD in the postoperative period. The use of CPB can cause deleterious effects associated with the systemic inflammatory response leading to lung injury and delayed recovery^[6]^. In contrast, OPCABG has been associated with better preservation of lung function, shorter MV time, and lower incidence of pulmonary complications^[6,24]^. These premises suggest that patients exposed to more lesions resulting from surgical techniques correlate with greater impairment in functional capacity.

Moreover, studies have also indicated that enhancing respiratory muscle strength during the preoperative period can improve outcomes following cardiac surgery^[25-27]^, and it has been strongly correlated with functional capacity^[18,26]^. A better fit on respiratory muscle strength before CABG has been determined to reduce postoperative pulmonary complications and may be associated with a lower perception of dyspnea resulting in delayed development of diaphragmatic fatigue, increased ventilatory efficiency, and improved response in the submaximal test^[26,27]^. Our findings corroborate these assertions, revealing that the lower the preoperative respiratory muscle strength, the higher the functional capacity impairment in the postoperative period. These findings suggest that strategies to improve respiratory muscle function could prevent loss of functional capacity following surgery.

The variation in 6MWD between the preoperative and postoperative periods that carries clinical relevance has not been examined previously. To our knowledge, this was the first study to assess the ability of the fall in 6MWD in the early postoperative period relative to the preoperative period as a predictor of midterm outcomes following CABG. This current study identified that the percentage fall in 6MWD during postoperative period was valuable in predicting midterm adverse outcomes after CABG during outpatient period. Patients who achieved a cutoff value > 34.6% of percentage fall in 6MWD on POD5 (considering the baseline preoperative value as 100%), presented with worse outcomes within three months following CABG. This period is equivalent to the end of the acute phase following a cardiac event and the beginning of phase III of cardiac rehabilitation. This result is essential for screening patients at hospital discharge, aiming to improve inpatient rehabilitation techniques during the early postoperative phase and subsequent secondary outpatient prevention strategies. This information may provide the perioperative heart team, which can make use of this data as a guide for discharge planning and postoperative rehabilitation management. Since inspiratory muscle strength was an independent predictor for percentage fall in 6MWD following POD5, it would be considered that patients who achieved a decline > 34.6% in 6MWD in early postoperative should be referred to a supervised outpatient rehabilitation with inspiratory muscle training added to an aerobic exercise program.

### Limitations

Some limitations of the current study should be discussed. Patients included in the present study were recruited during the inpatient period before elective CABG and the final number of patients was restricted due to the exclusion criteria and institutional issues (*i.e.*, emergency surgery, COPD, and the number of available operating rooms). Moreover, all 6MWT was performed on POD5 to standardize the recovery status of patients. A fixed period of time is important for clinical practice to detect patients who most need rehabilitation, even during hospitalization. Despite this, 29% of patients interrupted the test and a significant standard deviation was observed, which may indicate that some patients are not yet prepared to perform the test on POD5. These findings suggest that patients who are unable to perform 6MWT on POD5 are more likely to evolve with adverse midterm postoperative outcomes and should urgently undergo individualized multimodal rehabilitation. Lastly, the vast majority of patients included in the current study were male, therefore extrapolation of these findings to females warrants further evaluation.

## CONCLUSION

In conclusion, a cutoff value of 34.6% in percentage fall of 6MWD on POD5 predicted poorer clinical outcomes within three months of follow-up after CABG. Additionally, CPB and preoperative inspiratory muscle strength were independent predictors of percentage fall of 6MWD in the postoperative period. These findings further support the clinical application of the 6MWD and propose an inpatient preventive strategy to guide clinical management over time.

**Table:** 

Authors’ Roles & Responsibilities	
HOP	Substantial contributions to the conception or design of the work; or the acquisition, analysis, or interpretation of data for the work; drafting the work or revising it critically for important intellectual content; agreement to be accountable for all aspects of the work in ensuring that questions related to the accuracy or integrity of any part of the work are appropriately investigated and resolved; final approval of the version to be published
WJG	Substantial contributions to the conception or design of the work; or the acquisition, analysis, or interpretation of data for the work; drafting the work or revising it critically for important intellectual content; agreement to be accountable for all aspects of the work in ensuring that questions related to the accuracy or integrity of any part of the work are appropriately investigated and resolved; final approval of the version to be published
ISR	Substantial contributions to the conception or design of the work; or the acquisition, analysis, or interpretation of data for the work; drafting the work or revising it critically for important intellectual content; agreement to be accountable for all aspects of the work in ensuring that questions related to the accuracy or integrity of any part of the work are appropriately investigated and resolved; final approval of the version to be published
MV	Substantial contributions to the conception or design of the work; or the acquisition, analysis, or interpretation of data for the work; drafting the work or revising it critically for important intellectual content; agreement to be accountable for all aspects of the work in ensuring that questions related to the accuracy or integrity of any part of the work are appropriately investigated and resolved; final approval of the version to be published
BCMG	Substantial contributions to the conception or design of the work; or the acquisition, analysis, or interpretation of data for the work; drafting the work or revising it critically for important intellectual content; agreement to be accountable for all aspects of the work in ensuring that questions related to the accuracy or integrity of any part of the work are appropriately investigated and resolved; final approval of the version to be published
NOM	Substantial contributions to the conception or design of the work; or the acquisition, analysis, or interpretation of data for the work; drafting the work or revising it critically for important intellectual content; agreement to be accountable for all aspects of the work in ensuring that questions related to the accuracy or integrity of any part of the work are appropriately investigated and resolved; final approval of the version to be published
CBB	Substantial contributions to the conception or design of the work; or the acquisition, analysis, or interpretation of data for the work; drafting the work or revising it critically for important intellectual content; agreement to be accountable for all aspects of the work in ensuring that questions related to the accuracy or integrity of any part of the work are appropriately investigated and resolved; final approval of the version to be published
ASC	Substantial contributions to the conception or design of the work; or the acquisition, analysis, or interpretation of data for the work; drafting the work or revising it critically for important intellectual content; agreement to be accountable for all aspects of the work in ensuring that questions related to the accuracy or integrity of any part of the work are appropriately investigated and resolved; final approval of the version to be published
TPP	Substantial contributions to the conception or design of the work; or the acquisition, analysis, or interpretation of data for the work; drafting the work or revising it critically for important intellectual content; agreement to be accountable for all aspects of the work in ensuring that questions related to the accuracy or integrity of any part of the work are appropriately investigated and resolved; final approval of the version to be published
GOS	Substantial contributions to the conception or design of the work; or the acquisition, analysis, or interpretation of data for the work; drafting the work or revising it critically for important intellectual content; agreement to be accountable for all aspects of the work in ensuring that questions related to the accuracy or integrity of any part of the work are appropriately investigated and resolved; final approval of the version to be published
IB	Substantial contributions to the conception or design of the work; or the acquisition, analysis, or interpretation of data for the work; drafting the work or revising it critically for important intellectual content; agreement to be accountable for all aspects of the work in ensuring that questions related to the accuracy or integrity of any part of the work are appropriately investigated and resolved; final approval of the version to be published
CCS	Substantial contributions to the conception or design of the work; or the acquisition, analysis, or interpretation of data for the work; drafting the work or revising it critically for important intellectual content; agreement to be accountable for all aspects of the work in ensuring that questions related to the accuracy or integrity of any part of the work are appropriately investigated and resolved; final approval of the version to be published
RSLM	Substantial contributions to the conception or design of the work; or the acquisition, analysis, or interpretation of data for the work; drafting the work or revising it critically for important intellectual content; agreement to be accountable for all aspects of the work in ensuring that questions related to the accuracy or integrity of any part of the work are appropriately investigated and resolved; final approval of the version to be published
JNRB	Substantial contributions to the conception or design of the work; or the acquisition, analysis, or interpretation of data for the work; drafting the work or revising it critically for important intellectual content; agreement to be accountable for all aspects of the work in ensuring that questions related to the accuracy or integrity of any part of the work are appropriately investigated and resolved; final approval of the version to be published
GFV	Substantial contributions to the conception or design of the work; or the acquisition, analysis, or interpretation of data for the work; drafting the work or revising it critically for important intellectual content; agreement to be accountable for all aspects of the work in ensuring that questions related to the accuracy or integrity of any part of the work are appropriately investigated and resolved; final approval of the version to be published
NAHJ	Substantial contributions to the conception or design of the work; or the acquisition, analysis, or interpretation of data for the work; drafting the work or revising it critically for important intellectual content; agreement to be accountable for all aspects of the work in ensuring that questions related to the accuracy or integrity of any part of the work are appropriately investigated and resolved; final approval of the version to be published
RA	Substantial contributions to the conception or design of the work; or the acquisition, analysis, or interpretation of data for the work; drafting the work or revising it critically for important intellectual content; agreement to be accountable for all aspects of the work in ensuring that questions related to the accuracy or integrity of any part of the work are appropriately investigated and resolved; final approval of the version to be published
SG	Substantial contributions to the conception or design of the work; or the acquisition, analysis, or interpretation of data for the work; drafting the work or revising it critically for important intellectual content; agreement to be accountable for all aspects of the work in ensuring that questions related to the accuracy or integrity of any part of the work are appropriately investigated and resolved; final approval of the version to be published

## References

[1] Lawton JS, Tamis-Holland JE, Bangalore S, Bates ER, Beckie TM, Bischoff JM (2022). 2021 ACC/AHA/SCAI guideline for coronary artery
revascularization: executive summary: a report of the American college of
cardiology/American heart association joint committee on clinical practice
guidelines. Circulation.

[2] Thuijs DJFM, Kappetein AP, Serruys PW, Mohr FW, Morice MC, Mack MJ (2019). Percutaneous coronary intervention versus coronary artery bypass
grafting in patients with three-vessel or left main coronary artery disease:
10-year follow-up of the multicentre randomised controlled SYNTAX
trial. Lancet.

[3] Neumann FJ, Sousa-Uva M, Ahlsson A, Alfonso F, Banning AP, Benedetto U (2019). 2018 ESC/EACTS guidelines on myocardial
revascularization. Eur Heart J.

[4] Wynne R, Botti M (2004). Postoperative pulmonary dysfunction in adults after cardiac
surgery with cardiopulmonary bypass: clinical significance and implications
for practice. Am J Crit Care.

[5] Fiorina C, Vizzardi E, Lorusso R, Maggio M, De Cicco G, Nodari S (2007). The 6-min walking test early after cardiac surgery. Reference
values and the effects of rehabilitation programme. Eur J Cardiothorac Surg.

[6] Badenes R, Lozano A, Belda FJ (2015). Postoperative pulmonary dysfunction and mechanical ventilation in
cardiac surgery. Crit Care Res Pract.

[7] ATS Committee on Proficiency Standards for Clinical Pulmonary
Function Laboratories (2002). ATS statement: guidelines for the six-minute walk
test. Am J Respir Crit Care Med.

[8] Hirschhorn AD, Richards D, Mungovan SF, Morris NR, Adams L (2008). Supervised moderate intensity exercise improves distance walked
at hospital discharge following coronary artery bypass graft surgery--a
randomised controlled trial. Heart Lung Circ.

[9] Hirschhorn AD, Richards DA, Mungovan SF, Morris NR, Adams L (2012). Does the mode of exercise influence recovery of functional
capacity in the early postoperative period after coronary artery bypass
graft surgery? A randomized controlled trial. Interact Cardiovasc Thorac Surg.

[10] Guizilini S, Alves DF, Bolzan DW, Cancio AS, Regenga MM, Moreira RS, Trimer R (2014). Sub-xyphoid pleural drain as a determinant of functional capacity
and clinical results after off-pump coronary artery bypass surgery: a
randomized clinical trial. Interact Cardiovasc Thorac Surg.

[11] Bolzan DW, Trimer R, Begot I, Nasrala ML, Forestieri P, Mendez VM (2016). Open-lung ventilation improves clinical outcomes in off-pump
coronary artery bypass surgery: a randomized controlled
trial. J Cardiothorac Vasc Anesth.

[12] Standardization of Spirometry, 1994 Update (1995). American Thoracic Society. Am J Respir Crit Care Med.

[13] Soaresa MR, Pereira CA (2011). Six-minute walk test: reference values for healthy adults in
Brazil. J Bras Pneumol.

[14] American Thoracic Society/European Respiratory Society (2002). ATS/ERS statement on respiratory muscle testing. Am J Respir Crit Care Med.

[15] Neder JA, Andreoni S, Lerario MC, Nery LE (1999). Reference values for lung function tests. II. Maximal respiratory
pressures and voluntary ventilation. Braz J Med Biol Res.

[16] Zielińska D, Bellwon J, Rynkiewicz A, Elkady MA (2013). Prognostic value of the six-minute walk test in heart failure
patients undergoing cardiac surgery: a literature review. Rehabil Res Pract.

[17] De Feo S, Tramarin R, Lorusso R, Faggiano P (2009). Six-minute walking test after cardiac surgery: instructions for
an appropriate use. Eur J Cardiovasc Prev Rehabil.

[18] Stein R, Maia CP, Silveira AD, Chiappa GR, Myers J, Ribeiro JP (2009). Inspiratory muscle strength as a determinant of functional
capacity early after coronary artery bypass graft surgery. Arch Phys Med Rehabil.

[19] Cordeiro AL, de Melo TA, Neves D, Luna J, Esquivel MS, Guimarães AR (2016). Inspiratory muscle training and functional capacity in patients
undergoing cardiac surgery. Braz J Cardiovasc Surg.

[20] Bohmer RM, Newell J, Torchiana DF (2002). The effect of decreasing length of stay on discharge destination
and readmission after coronary bypass operation. Surgery.

[21] Cowper PA, DeLong ER, Hannan EL, Muhlbaier LH, Lytle BL, Jones RH (2007). Is early too early? Effect of shorter stays after bypass
surgery. Ann Thorac Surg.

[22] Almashrafi A, Alsabti H, Mukaddirov M, Balan B, Aylin P (2016). Factors associated with prolonged length of stay following
cardiac surgery in a major referral hospital in Oman: a retrospective
observational study. BMJ Open.

[23] Guizilini S, Gomes WJ, Faresin SM, Bolzan DW, Buffolo E, Carvalho AC (2007). De Influence of pleurotomy on pulmonary function after off-pump
coronary artery bypass grafting. Ann Thorac Surg.

[24] Guizilini S, Gomes WJ, Faresin SM, Bolzan DW, Alves FA, Catani R (2005). Evaluation of pulmonary function in patients following on and
off-pump coronary artery bypass grafting. Braz J Cardiovasc Surg.

[25] Hulzebos EH, Helders PJ, Favié NJ, De Bie RA, Brutel de la Riviere A, Van Meeteren NL (2006). Preoperative intensive inspiratory muscle training to prevent
postoperative pulmonary complications in high-risk patients undergoing CABG
surgery: a randomized clinical trial. JAMA.

[26] Hulzebos EH, Smit Y, Helders PP, van Meeteren NL (2012). Preoperative physical therapy for elective cardiac surgery
patients. Cochrane Database Syst Rev.

[27] Katsura M, Kuriyama A, Takeshima T, Fukuhara S, Furukawa TA (2015). Preoperative inspiratory muscle training for postoperative
pulmonary complications in adults undergoing cardiac and major abdominal
surgery. Cochrane Database Syst Rev.

